# Coronary microvascular dysfunction is associated with degree of anaemia in end‐stage renal disease

**DOI:** 10.1186/s12872-021-02025-2

**Published:** 2021-04-26

**Authors:** Ashwin Radhakrishnan, Luke C. Pickup, Anna M. Price, Jonathan P. Law, Kirsty C. McGee, Larissa Fabritz, Roxy Senior, Richard P. Steeds, Charles J. Ferro, Jonathan N. Townend

**Affiliations:** 1 Birmingham Cardio-Renal Group, Institute of Cardiovascular Sciences, University of Birmingham, Birmingham, United Kingdom; 2 Department of Cardiology, Queen Elizabeth Hospital, Birmingham, United Kingdom; 3 Department of Nephrology, Queen Elizabeth Hospital, Birmingham, United Kingdom; 4 Institute of Inflammation and Ageing, University of Birmingham, Birmingham, United Kingdom; 5 Institute of Cardiovascular Sciences, University of Birmingham, Birmingham, United Kingdom; 6 Cardiac Research Unit, Northwick Park Hospital, London, United Kingdom; 7 Department of Cardiology, Royal Brompton Hospital, London, United Kingdom

**Keywords:** Coronary flow velocity reserve, Anaemia, End-stage renal disease, Coronary microvascular dysfunction

## Abstract

**Background:**

Coronary microvascular dysfunction (CMD) is common in end-stage renal disease (ESRD) and is an adverse prognostic marker. Coronary flow velocity reserve (CFVR) is a measure of coronary microvascular function and can be assessed using Doppler echocardiography. Reduced CFVR in ESRD has been attributed to factors such as diabetes, hypertension and left ventricular hypertrophy. The contributory role of other mediators important in the development of cardiovascular disease in ESRD has not been studied. The aim of this study was to examine the prevalence of CMD in a cohort of kidney transplant candidates and to look for associations of CMD with markers of anaemia, bone mineral metabolism and chronic inflammation.

**Methods:**

Twenty-two kidney transplant candidates with ESRD were studied with myocardial contrast echocardiography, Doppler CFVR assessment and serum multiplex immunoassay analysis. Individuals with diabetes, uncontrolled hypertension or ischaemic heart disease were excluded.

**Results:**

7/22 subjects had CMD (defined as CFVR < 2). Demographic, laboratory and echocardiographic parameters and serum biomarkers were similar between subjects with and without CMD. Subjects with CMD had significantly lower haemoglobin than subjects without CMD (102 g/L ± 12 vs. 117 g/L ± 11, p = 0.008). There was a positive correlation between haemoglobin and CFVR (r = 0.7, p = 0.001). Similar results were seen for haematocrit. In regression analyses, haemoglobin was an independent predictor of CFVR (β = 0.041 95% confidence interval 0.012–0.071, p = 0.009) and of CFVR < 2 (odds ratio 0.85 95% confidence interval 0.74–0.98, p = 0.022).

**Conclusions:**

Among kidney transplant candidates with ESRD, there is a high prevalence of CMD, despite the absence of traditional risk factors. Anaemia may be a potential driver of microvascular dysfunction in this population and requires further investigation.

## Background

Coronary microvascular dysfunction (CMD) is common among patients with chronic kidney disease (CKD) [1]. With each increase in CKD stage, there is a corresponding rise in rates of CMD, with the highest prevalence among patients with end-stage renal disease (ESRD) [2, 3]. The presence of CMD is a poor prognostic marker and may partly explain the excessive cardiac risk associated with CKD [3–5]. Coronary flow velocity reserve (CFVR) is a recognised measure of microvascular function. It reflects the ability of the coronary microcirculation to respond to vasodilatory stimuli and can be reliably detected using Doppler transthoracic echocardiography [6]. In individuals with normal coronary microvascular function, coronary flow should at least double at maximal hyperaemia. Therefore, CFVR < 2, in the absence of obstructive coronary artery disease (CAD), is widely accepted to signify CMD [1].

The syndrome of uraemic cardiomyopathy, characterised by left ventricular hypertrophy (LVH), diffuse interstitial fibrosis, systolic and diastolic dysfunction and an increased risk of sudden cardiac death, represents advanced cardiac disease in ESRD, and is associated with significantly worse cardiovascular outcomes [7, 8]. Factors such as diabetes and hypertension, that contribute to the development of uraemic cardiomyopathy, have been linked with CMD in CKD [9, 10]. A number of other mediators, including anaemia, bone mineral disease and chronic inflammation, are important in the aetiology of uraemic cardiomyopathy. Their impact on the development of CMD in ESRD remain unknown. The aim of this hypothesis generating study was to examine the prevalence of CMD among a population of potential kidney transplant recipients, and to look for associations between CMD and markers of anaemia, bone mineral disease and chronic inflammation.

## Methods

### Study population

Twenty-two kidney transplant candidates with ESRD who successfully underwent CFVR assessment at the Queen Elizabeth Hospital, Birmingham (QEHB), United Kingdom between March 2019 and March 2020 were included in this analysis. These individuals were research participants in the Chronic Renal Impairment in Birmingham Coronary Flow Reserve (CRIB-FLOW) study or the Prospective Study of the Effects of Renal Transplantation on Uraemic Cardiomyopathy using Magnetic Resonance Imaging (RETRACT) echocardiogram sub-study, both of which examined CFVR in patients with ESRD.

Participants were > 18 years old, considered suitable for kidney transplantation by the renal transplant team at QEHB, had estimated glomerular filtration rate (eGFR) < 15 ml/min/1.73 m^2^ and were pre-dialysis or on peritoneal dialysis (PD). Exclusion criteria were: pregnancy, haemodialysis (HD), diabetes mellitus, uncontrolled hypertension, known ischaemic heart disease, moderate/severe valvular heart disease and contraindication to adenosine or sulphur hexafluoride contrast agent (SonoVue, Bracco, Milan, Italy).

### Blood pressure

Office blood pressure (BP) was measured using an automated BP monitor (BpTRU, VSM Medtech, Coquitlam, BC, Canada), which takes 6 BP readings over 6 min. After exclusion of the first reading, an average of the remaining 5 readings was used to represent office BP.

### Transthoracic echocardiography (TTE)

All subjects underwent comprehensive two-dimensional echocardiography by a British Society of Echocardiography accredited physician (AR). Studies were performed on a Philips iE33 machine (Philips, Eindhoven, Netherlands) using a S5-1 transducer for TTE and myocardial contrast echocardiogram (MCE) studies and a S8-3 transducer for CFVR measurements. Echocardiograms were stored under an anonymous code and analysed offline by a single investigator (AR) using commercially available software (IntelliSpace Cardiovascular, Philips, Eindhoven, Netherlands).

Left ventricular mass was estimated using the Cube formula and indexed for body surface area [11]. The Simpson’s biplane method was used to measure left ventricular volumes and ejection fraction [11]. Diastolic function was quantified using multiple parameters [12]. Global longitudinal strain (GLS) was assessed in the 3 standard apical views using speckle tracking.

### Doppler coronary flow velocity reserve

Doppler CFVR assessment was performed as previously described [13]. The left anterior descending artery (LAD) was identified on colour Doppler in the anterior inter-ventricular sulcus. Pulse wave Doppler signals of LAD flow were recorded to measure coronary flow velocity (CFV) at rest and at hyperaemia. SonoVue was used, if necessary, to identify LAD flow and to improve the spectral Doppler trace. Hyperaemia was induced by an infusion of adenosine at a rate of 140micrograms/kg/min for 3 min. Subjects were advised to abstain from caffeine for 24 h prior to adenosine administration. CFVR was calculated as hyperaemic CFV/rest CFV. For each variable in the CFVR calculation, the highest values of 3 cardiac cycles were averaged.

### Myocardial contrast echocardiography

Myocardial contrast echocardiography was performed as previously described [13]. Images were taken in the 3 apical views using low-power continuous MCE at a mechanical index (MI) of 0.1. Sonovue was continually infused using an oscillating infusion pump that maintains microbubbles in suspension (Vueject, Bracco, Milan, Italy). The infusion rate was started at 70-100ml/hr but adjusted to ensure sufficient myocardial opacification without excessive contrast attenuation. Triggered high MI (1.0) flash echocardiography was performed at end-systole, where the myocardium is at its thickest, to destroy microbubbles in the myocardium and to observe replenishment. The sequence was initially performed at rest and then repeated after adenosine vasodilator stress as above. The absence of regional wall motion abnormalities or sub-endocardial perfusion defects on vasodilator MCE was deemed sufficient to exclude flow limiting CAD.

### Laboratory analysis

N-terminal pro-brain natriuretic peptide (NTpro-BNP) was assayed using the Alere point of care assay (Alere, Massachusetts, USA). High sensitivity C-reactive peptide was assayed using the Architect MULTIGENT CRP Vario assay (Abbott, Illinois, USA). The remaining laboratory parameters were assayed using standardised automated methods. The fluorescence responses of 16-analytes of inflammation, atrial stretch, cardiac fibrosis, kidney injury and LVH were obtained using Human Magnetic Luminex® Asssays (R&D Systems, Minneapolis, MN, USA) and the Bio-RAD Bio-Plex™ 200 system for analysis. Concentrations were calculated using the Bio-Plex Software Manager™ (version 6.1) generated standard curves and a 5PL logistic curve fitting technique as per the manufacturer’s instructions.

### Statistical analysis

Statistical analysis was performed using SPSS version 26 (SPSS Inc, Chicago, Illinois). The Shapiro–Wilk test was used to assess data normality. Continuous variables are expressed as mean ± standard deviation for parametric data or median (interquartile range—IQR) for non-parametric data. Unpaired group comparisons for continuous data were made using the unpaired t-test or the Mann-Whitney U test. Unpaired categorical data were compared using Fisher’s exact test. Correlation was assessed using the Pearson correlation coefficient. Univariable and multivariable linear regression models were performed with CFVR as the dependent variable. Factors known to influence CFVR (age, systolic BP, left ventricular mass index) as well as markers of anaemia (haemoglobin, iron), bone mineral disease [calcium, phosphate, parathyroid hormone (PTH)] and inflammation (high sensitivity C-reactive peptide, tumour necrosis factor-α, interleukin-6, interleukin-8, interleukin-10) were included as independent variables in regression models. Binary logistic regression was also performed, with CFVR < 2 as the dependent variable, and the parameters listed above as independent variables. Parameters that were significant in univariable analysis were entered into multivariable regression models. A variance inflation factor > 5 was taken to represent collinearity. Statistical tests were 2-tailed, and a p value < 0.05 was considered statistically significant.

## Results

### Subject characteristics

Twenty-two kidney transplant candidates with ESRD (8 pre-dialysis and 14 PD) were included. The aetiology of ESRD was: glomerulonephritis (45%), polycystic kidney disease (23%), hypertension (9%), obstructive uropathy (9%), pyelonephritis (9%) and idiopathic (5%). No participants had symptoms of ischaemic heart disease or heart failure at study enrolment. 14/22 (64%) had undergone prior cardiovascular assessment for CAD as part of the transplant recipient cardiac work-up protocol at the Queen Elizabeth Hospital, Birmingham using myocardial perfusion scintigraphy (n = 11), exercise stress echocardiography (n = 2) or invasive coronary angiography (n = 1). Median time from cardiovascular assessment to study enrolment for these individuals was 18 months (IQR 3–33 months). The remaining 8 participants did not require cardiovascular assessment as per our institutional protocol.

Using CFVR < 2 to signify CMD, 7/22 (32%) of our cohort with ESRD had CMD. Mean CFVR for subjects with CMD was 1.6 ± 0.2. Mean CFVR for subjects without CMD was 3.2 ± 0.9. Previously published data by our group demonstrated a reference value of CFVR in healthy controls of 3.8 ± 0.6 [13]. Baseline demographic, laboratory and haemodynamic data for subjects with and without CMD are shown in Table 1. There were no significant demographic or haemodynamic differences between the 2 groups. There were similar numbers of PD patients in both groups. Hypertension and hypercholesterolaemia (defined as total cholesterol > 5 mmol/L or statin therapy) were common in the entire cohort, but the prevalence of these comorbidities was not significantly higher in subjects with CFVR < 2.

**Table 1:** Demographic, laboratory and haemodynamic variables

	CFVR < 2 (n = 7)	CFVR ≥ 2 (n = 15)	p value
Demographics			
Age (years)	47 ± 15	55 ± 10	0.177
Male n (%)	3 (43)	8 (53)	1.0
Caucasian n (%)	5 (71)	12 (80)	1.0
BMI (kg/m^2^)	26.3 ± 4.4	27.7 ± 4.9	0.527
Smoker n (%)—Ex Never Current	1 (14) 6 (86) 0 (0)	4 (27) 10 (67) 1 (6)	0.744
Hypertension n (%)	6 (86)	14 (93)	1.0
Hypercholesterolaemia n (%)	4 (57)	11 (73)	0.630
Peritoneal dialysis n (%)	5 (71)	9 (60)	1.0
Duration of dialysis (months)	5 (4–48)	6 (4–9)	0.797
ACE inhibitor n (%)	1 (14)	4 (27)	1.0
ARB n (%)	1 (14)	3 (20)	1.0
Statin n (%)	1 (14)	8 (53)	0.165
Loop diuretic n (%)	5 (71)	5 (33)	0.172
Calcium channel blocker n (%)	5 (71)	9 (60)	1.0
Beta blocker n (%)	2 (29)	3 (20)	1.0
Alpha blocker	3 (43)	4 (27)	0.630
Erythropoietin treatment n (%)	5 (71)	4 (27)	0.074
Laboratory data			
** Haemoglobin (g/L)**	**102 ± 12**	**117 ± 11**	**0.008**
** Haematocrit (%)**	**31.2 ± 3.3**	**35.4 ± 3.7**	**0.019**
Mean cell volume (fl.)	88.9 ± 3.3	91.6 ± 3.7	0.118
Urea (mmol/L)	21.8 ± 6.2	22.1 ± 5.6	0.902
Creatinine (µmol/L)	673 ± 300	606 ± 192	0.534
ACR (mg/mmol)	204 (109.3-277.8)	77.4 (62.8-199.4)	0.239
Iron (µmol/L)	11.8 (9.5–13)	12.9 (9.4–16)	0.494
Transferrin (g/L)	1.92 ± 0.54	2.06 ± 0.4	0.525
Albumin (g/L)	35 ± 6	40 ± 7	0.125
Corrected calcium (mmol/L)	2.45 ± 0.13	2.33 ± 0.17	0.123
hsCRP (mg/L)	1.9 (1-3.6)	2.8 (1.9-8)	0.312
NT pro-BNP (ng/L)	1900 (522–4597)	441 (342–643)	0.416
Phosphate (mmol/L)	1.71 (1.55–2.07)	1.59 (1.53–1.69)	0.312
PTH (µmol/L)	41.7 ± 23.2	30.5 ± 16.9	0.271
Total cholesterol (mmol/L)	4.8 ± 1.7	5.0 ± 1.4	0.772
Haemodynamic data			
Systolic BP (mmHg)	129 ± 25	137 ± 20	0.398
Diastolic BP (mmHg)	83 ± 14	85 ± 8	0.798
Heart Rate (bpm)	72 ± 14	66 ± 8	0.156

Data are presented as mean ± SD or median (IQR). Variables highlighted in bold demonstrated a significant difference between the two groups

CFVR, coronary flow velocity reserve; BMI, body mass index; ACE, angiotensin converting enzyme; ARB, angiotensin receptor blocker; ACR, albumin creatinine ratio; hsCRP, high sensitivity C reactive peptide; NT-proBNP, N terminal pro brain natriuretic peptide; PTH, parathyroid hormone; BP, blood pressure; bpm, beats per minute

### Anaemia

**Fig. 1:**
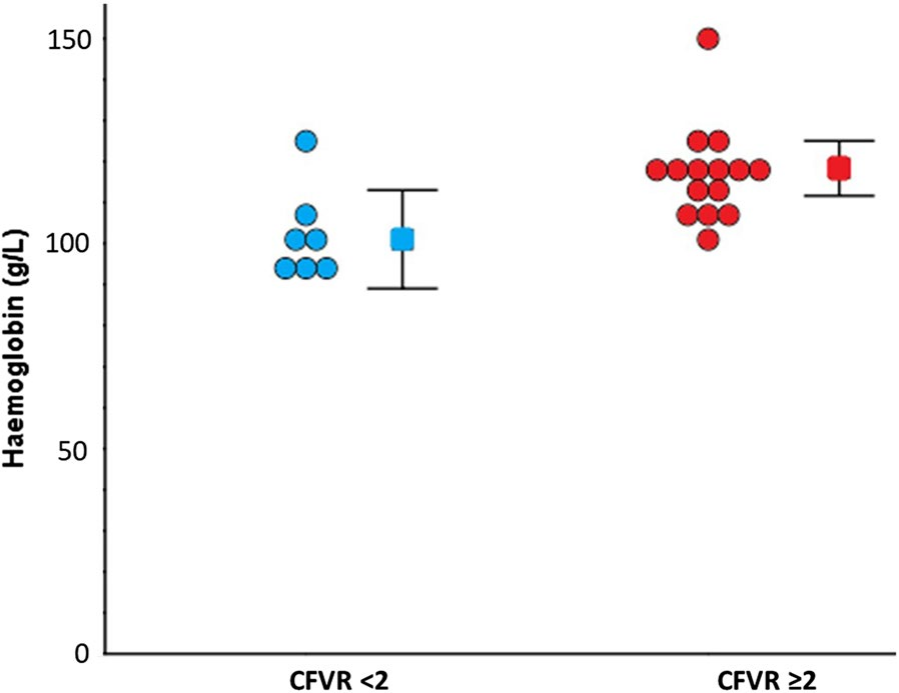
Haemoglobin in subjects with CFVR < 2 and CFVR ≥ 2. Circles represent individual measurements. Squares represent mean. Error bars represent 95% confidence intervals of the mean. CFVR, coronary flow velocity reserve

**Fig. 2:**
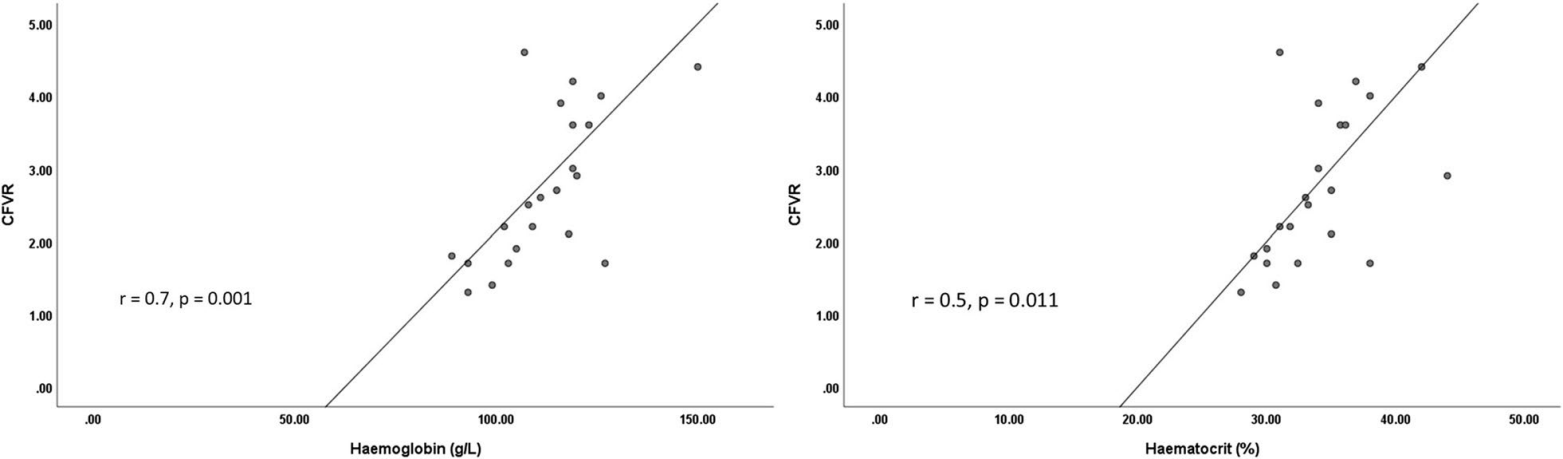
Correlation between coronary flow velocity reserve and haemoglobin (left) and haematocrit (right). CFVR, coronary flow velocity reserve

Anaemia (defined as < 120 g/L in females and < 130 g/L in males) [14] was present in 17/22 (77%) of the whole cohort, and was normocytic in all cases. Haemoglobin concentration was significantly lower in patients with CMD compared to those without CMD [102 g/L ± 12 vs. 117 g/L ± 11, mean difference 15 g/L, 95% confidence interval (CI) 4–26, p = 0.008]—Fig. 1. There was a corresponding significantly lower haematocrit among subjects with CMD (31.2% ± 3.1 vs. 35.4% ± 3.7, mean difference 4.2%, 95% CI 0.8–7.8, p = 0.019). There were positive correlations between CFVR and haemoglobin (r = 0.7, p = 0.001) and between CFVR and haematocrit (r = 0.5, p = 0.011)—Fig. 2.

### Bone mineral disease

Markers of CKD bone mineral disease were similar between the two groups. Calcium, phosphate and PTH were all numerically higher in patients with CMD, but this was not statistically significant.

### Inflammatory markers

One subject with CMD did not provide stored blood for serum multiplex immunoassay. Inflammatory markers were similar among subjects with CMD and those with normal coronary microvascular function—Table 2. Analysis of the remaining biomarkers studied by multiplex immunoassay also did not show any significant differences between the two groups.

**Table 2:** Results of human magnetic luminex assay

Assay	CFVR < 2 (n = 6)	CFVR ≥ 2 (n = 15)	p value
Angiopoetin-2 (pg/ml)	3274 (1000–5136)	3051 (2230–4053)	0.850
Atrial natriuretic peptide (pg/ml)	25,836 ± 9520	20,568 ± 11,210	0.329
Detectable KIM-1 n (%)	2 (33)	5 (31)	1.0
Galectin-3 (ng/ml)	1.3 (1-1.3)	1.3 (1-1.4)	0.791
IL-1ra (pg/ml)	667 (526–742)	515 (384–729)	0.850
IL-6 (pg/ml)	2.09 ± 1.3	2.69 ± 1.35	0.371
IL-8 (pg/ml)	6.1 (4.2–11.5)	11.4 (8–23)	0.132
IL-10 (pg/ml)	2.5 (0.9–4.1)	1.4 (0.9–3.4)	1.0
Leptin (ng/ml)	17.7 (6.6–20.6)	13.2 (4.2–50.4)	0.910
MCP-1 (pg/ml)	396 ± 221	375 ± 102	0.770
MMP-9 (pg/ml)	10,614 (4955–11,509)	9880 (6244–13,648)	1.0
NGAL (ng/ml)	26.3 ± 8.9	26.6 ± 4.8	0.898
ST2 (ng/ml)	14 (10–33)	12 (9–19)	0.850
TNF${\upalpha }$ (pg/ml)	6.1 (4.5–8.1)	5.7 (5.1–6.7)	0.850
Uromodulin (ng/ml)	18 ± 9	21 ± 10	0.53
VEGF (pg/ml)	52 ± 26	75 ± 25	0.108

Data are presented as mean ± SD or median (IQR)

CFVR, coronary flow velocity reserve; KIM-1, kidney injury molecule 1; IL-1ra, interleukin 1 receptor antagonist; IL-6, interleukin-6; IL-8, interleukin-8; IL-10, interleukin-10; MCP-1, monocyte chemoattractant protein; MMP-9, matrix metallopeptidase 9; NGAL, neutrophil gelatinase associated lipocalin; ST2, suppression of tumorigenicity 2; TNFα, tumour necrosis factor alpha; VEGF, vascular endothelial growth factor.

### Echocardiographic data

Echocardiographic data are reported in Table 3. Left ventricular dimensions, mass index and systolic and diastolic function were similar between the two groups. Cardiac output was significantly higher in subjects with CMD (6.1 L/min ± 0.8 vs. 4.7 L/min ± 1.4, mean difference 1.4 L/min, 95% CI 0.3-2.5 L/min, p = 0.02). No subjects had regional wall motion abnormalities or perfusion defects on MCE.

**Table 3:** Echocardiographic parameters

	CFVR < 2 (n = 7)	CFVR ≥ 2 (n = 15)	p value
IVSD (mm)	12 ± 1	11 ± 2	0.610
LVIDD (mm)	46 ± 9	47 ± 6	0.679
PWD (mm)	10 ± 2	11 ± 2	0.789
LVIDS (mm)	31 (29–36)	30 (28–35)	0.535
FS (%)	33 ± 9	35 ± 5	0.639
LVEDVi (ml/m^2^)	55 (49–69)	44 (39–51)	0.115
LVESVi (ml/m^2^)	21 (18–28)	18 (16–21)	0.275
EF (%)	59 ± 7	59 ± 4	0.923
Stroke volume (ml)	87 ± 25	72 ± 20	0.182
**Cardiac output (L/min)**	**6.1 ± 0.8**	**4.7 ± 1.4**	**0.02**
GLS (%)	-16 ± 3	-19 ± 2	0.107
TAPSE (mm)	21 ± 4	21 ± 5	0.875
LV mass index (g/m^2^)	99 ± 31	98 ± 28	0.936
LV geometry n (%)—normal geometry Concentric remodelling Eccentric hypertrophy Concentric hypertrophy	2 (29) 3 (43) 1 (14) 1 (14)	4 (27) 1 (7) 3 (20) 7 (46)	0.237
LA volume index (ml/m^2^)	31.3 (26-44.1)	28.8 (20-38.3)	0.630
E/A ratio	1.1 (0.9–1.2)	0.8 (0.7–1.1)	0.340
E/e′	9 (8–11)	8 (7–10)	0.123

Data are presented as mean ± SD or median (IQR) .Variables highlighted in bold demonstrated a significant difference between the groups

CFVR, coronary flow velocity reserve; IVSD, interventricular septal diameter; LVIDD, left ventricular internal diameter diastole; PWD, posterior wall diameter; LVIDS, left ventricular internal diameter systole; LVEDVi, indexed left ventricular end diastolic volume; LVESVi, indexed left ventricular end systolic volume; EF, ejection fraction; GLS, global longitudinal strain; TAPSE, tricuspid annular plane systolic excursion; LV, left ventricular.

### Regression analysis

In univariable linear regression analysis, haemoglobin and iron were independent predictors of CFVR—haemoglobin (β = 0.051 95% CI 0.023–0.079, p = 0.001) and iron (β = 0.094 95% CI 0.003–0.185, p = 0.044). However, in multivariable analysis, only haemoglobin was an independent predictor of CFVR (β = 0.041 95% CI 0.012–0.071, p = 0.009). In univariable binary logistic regression, haemoglobin was a negative predictor of CFVR < 2 (Odds ratio 0.85 95% CI 0.74–0.98, p = 0.022). No other parameters showed a significant association with CFVR < 2.

## Discussion

This study has confirmed a high prevalence of CMD in subjects with ESRD. To our knowledge, it is also the first study to suggest an association between CMD and anaemia in this population. It is recognised that patients on the kidney transplant waiting list are often younger, have fewer comorbidities, and a reduced risk of death compared to ESRD patients not suitable for kidney transplantation [15]. However, previous work has shown that CMD was present in 59% of patients with ESRD undergoing evaluation for kidney transplant, and was more common in those with diabetes or left ventricular systolic dysfunction [16]. Unlike this study, our cohort did not include individuals with diabetes and uncontrolled hypertension, both of which independently influence CFVR [9, 10]. Despite this, nearly a third of our cohort of potential kidney transplant candidates had CFVR < 2.

The presence of anaemia in patients with CKD is associated with a significantly increased risk of cardiovascular and all-cause mortality [17]. Thus, our novel finding that subjects with ESRD and CMD have lower haemoglobin than patients with normal CFVR raises the possibility that this adverse association with prognosis may be in part related to the presence of CMD. Despite comparable kidney function and iron stores, and a higher prevalence of erythropoietin treatment, subjects with CMD had significantly lower haemoglobin and haematocrit than those with CFVR ≥ 2. We have also shown an association between haemoglobin and CFVR, that is independent of traditional factors thought to influence CFVR such as hypertension, diabetes and left ventricular hypertrophy. As anaemia is extremely prevalent in ESRD, low haemoglobin maybe an important driver of microvascular dysfunction and the increased cardiovascular mortality seen in this population. Furthermore, patients with CKD have additional risk factors for CMD, which may be exacerbated by anaemia.

These findings are of potential importance. While we cannot assume causation in either direction, it seems unlikely that anaemia could be caused by CMD. Our findings are also unlikely to be related to the methodology of our imaging technique since measurement of CFVR by Doppler TTE is not conventionally adjusted for haemoglobin as the pulse wave Doppler velocity signal is independent of haemoglobin concentration [18]. There are biologically plausible reasons why anaemia may lead to CMD in ESRD. Anaemia causes a number of maladaptive changes to the cardiovascular system that may predispose to CMD. Chronic anaemia can induce a form of high output cardiac failure, that leads to adverse cardiac remodelling including left ventricular dilatation, volume overload and LVH [19, 20]. This is suggested in our cohort, where subjects with CMD had an increased cardiac output, as well as a trend towards increased left ventricular and atrial volumes and markers of myocardial stretch. Animal studies in anaemia have shown that in order to maintain adequate myocardial oxygen supply, there is an increase in resting myocardial blood flow compared to non-anaemic controls, predominantly due to capillary widening and reduced blood viscosity [20]. Thus, in anaemia, the microcirculation operates in a state of supra-normal vasodilation at rest, which may limit its ability to vasodilate further during hyperaemia. Anaemia is also associated with abnormal red cell function and reduced nitric oxide bioactivity, which further impairs endothelium-dependent vasodilation in the microcirculation [21]. It is plausible that the combination of increased basal myocardial blood flow and a submaximal hyperaemic response leads to reduced CFVR in conditions of chronic anaemia—a pattern seen among our subjects with CMD.

Alternatively, a common causative factor may result in both anaemia and CMD. Possibilities include systemic inflammation and malnutrition, which are both commonly found in chronic disease states. We found no strong evidence that patients with CMD had higher levels of inflammatory markers. Markers of nutritional status such as body mass index, albumin and cholesterol were numerically lower among subjects in our cohort with CMD, but this was not statistically significant. It is possible that the small sample size means that we were unable to detect subtle differences in these variables.

To date there are no other studies examining the effect of anaemia on CFVR in CKD. However, there is some evidence from other conditions of an association between anaemia and CMD. In patients with beta thalassemia minor, Doppler CFVR was significantly lower compared to control subjects matched for age, gender and BMI [22]. Similarly, in patients with sickle cell disease, studies have demonstrated impaired coronary microvascular function compared to healthy controls. However, the aetiology of CMD in sickle cell disease is likely to be different to that seen in CKD and may be related to microvascular obstruction from vaso-occlusive events [23, 24]. A single study also included a group of patients with iron deficiency anaemia but did not demonstrate any reduction in CFVR compared to healthy controls [23].

The clinical significance of our findings requires further investigation. Current guidelines recommend aiming for a haemoglobin concentration > 90 g/L in patients on dialysis and > 100 g/L in non-dialysis CKD patients [14]. We have demonstrated that significant reductions in CFVR are present even above these treatment thresholds. Previous studies of aggressive anaemia treatment in CKD have had disappointing results, with no improvement in cardiovascular outcomes and possibly an increased risk of harm from correcting haemoglobin to a higher threshold [25]. To date, there are no studies examining the impact of improving haemoglobin concentration on CFVR.

## Limitations

The main limitation of our study is the small sample size, which was limited by the outbreak of the global COVID-19 pandemic. Despite the small sample size, we found a high prevalence of CMD among this cohort. Furthermore, the size of the difference in haemoglobin and the strength of the relationship between haemoglobin and CFVR in multivariable analysis suggests that this is a true finding. Our study was underpowered to find small differences in the other variables tested.

Similar to other non-invasive studies of CFVR, we could not fully exclude occult CAD among our population. However, the majority of subjects in our study had undergone prior screening for CAD. Furthermore, all subjects were asymptomatic, had normal electrocardiograms and no coronary distribution perfusion defect or regional wall motion abnormality on vasodilator MCE—a highly sensitive and specific technique for the diagnosis of CAD [26]. This provides strong indirect evidence that there was no obstructive CAD in our cohort.

We included only patients eligible for kidney transplantation in this study. We also excluded patients on HD, as echocardiographic measurements in this population are more volume dependent [27]. These tight inclusion criteria improve the validity of our findings in the population we studied but limits the generalisability of our findings to the wider ESRD population.

Finally, our study was cross-sectional in design, meaning that causation cannot be definitively demonstrated. Future longitudinal work examining the role of anaemia and its correction on CFVR is needed.

## Conclusions

Among patients suitable for kidney transplantation, there is a high prevalence of CMD, even in the absence of traditional risk factors such as diabetes, uncontrolled hypertension or significant LVH. In this population, we have shown that CMD is associated with low haemoglobin and an increased cardiac output—findings that require further investigation and independent confirmation. Together, they suggest that anaemia is a possible driver of CMD in ESRD. If this association is confirmed in larger studies, then correction of anaemia may represent a potential therapeutic target to improve microvascular function in ESRD.

## Data Availability

The datasets used and analysed during the current study are available from the corresponding author on reasonable request.

## Abbreviations

BP: Blood pressure
CAD: Coronary artery disease
CFVR: Coronary flow velocity reserve
CFV: Coronary flow velocity
CI: Confidence interval
CKD: Chronic kidney disease
CMD: Coronary microvascular dysfunction
CRIB-FLOW: Chronic Renal Impairment in Birmingham Coronary FLOW Reserve study
eGFR: Estimated glomerular filtration rate
ESRD: End-stage renal disease
GLS: Global longitudinal strain
IQR: Inter-quartile range
LAD: Left anterior descending artery
MCE: Myocardial contrast echocardiogram
MI: Mechanical index
NTpro-BNP: N-terminal pro-brain natriuretic peptide
PTH: Parathyroid hormone
QEHB: Queen Elizabeth Hospital Birmingham
RETRACT: Prospective Study of the Effects of Renal Transplantation on Uraemic Cardiomyopathy using Magnetic Resonance Imaging
TTE: Transthoracic echocardiogram
